# Screening and Purification of Natural Products from Actinomycetes that Induce a “Rounded” Morphological Phenotype in Fission Yeast

**DOI:** 10.1007/s13659-021-00304-1

**Published:** 2021-04-21

**Authors:** Richard Alexander Lewis, Jenileima Devi, Katherine Green, Juanjuan Li, Adam Hopkins, Jacqueline Hayles, Paul Nurse, Jeff Errington, Nicholas Edward Ellis Allenby

**Affiliations:** 1 Demuris Ltd, The Biosphere, Draymans Way, Newcastle Helix, Newcastle upon Tyne, NE4 5BX UK; 2 University of Southampton, University Road, Southampton, SO17 1BJ UK; 3 Cell Cycle Laboratory, The Francis Crick Institute, 1 Midland Road, London, NW1 1AT UK

**Keywords:** Actinomycetes, *Schizosaccharomyces pombe*, Morphology, Phenotype, Antifungal, Polyene

## Abstract

This study was designed to identify and investigate bioactive natural product compounds that alter the cellular shape of the fission yeast *Schizosaccharomyces pombe* and induce a “rounded” or “small” cellular morphological phenotype. Bioassays using a range of antifungal agents against a multidrug-sensitive fission yeast strain, SAK950 showed that many induced a “rounded” phenotype. We then investigated whether 46 of the actinomycete strains identified in our previous study as inducing a similar phenotype produced antifungal agents of similar classes. We show that five of the strains produced streptothricin and that 26 strains produced polyenes, including fungichromin, filipin and candicidin, the last of which was produced by 24 strains. A taxonomic study of the strains indicated that the majority of the candicidin only producers were *Streptomyces hydrogenans* and *S. albidoflavus* whilst those that additionally produced streptothricin were related to *S. enissocaesilis*. A follow-up study to investigate the natural products made by related strains indicated that they followed a similar pattern. The identification of several compounds from the actinomycete strains similar to the antifungal agents initially tested confirm the validity of an approach using the *S. pombe* morphological phenotype and actinomycete taxonomy as a predictive tool for natural product identification.

**Supplementary Information:**

The online version contains supplementary material available at 10.1007/s13659-021-00304-1.

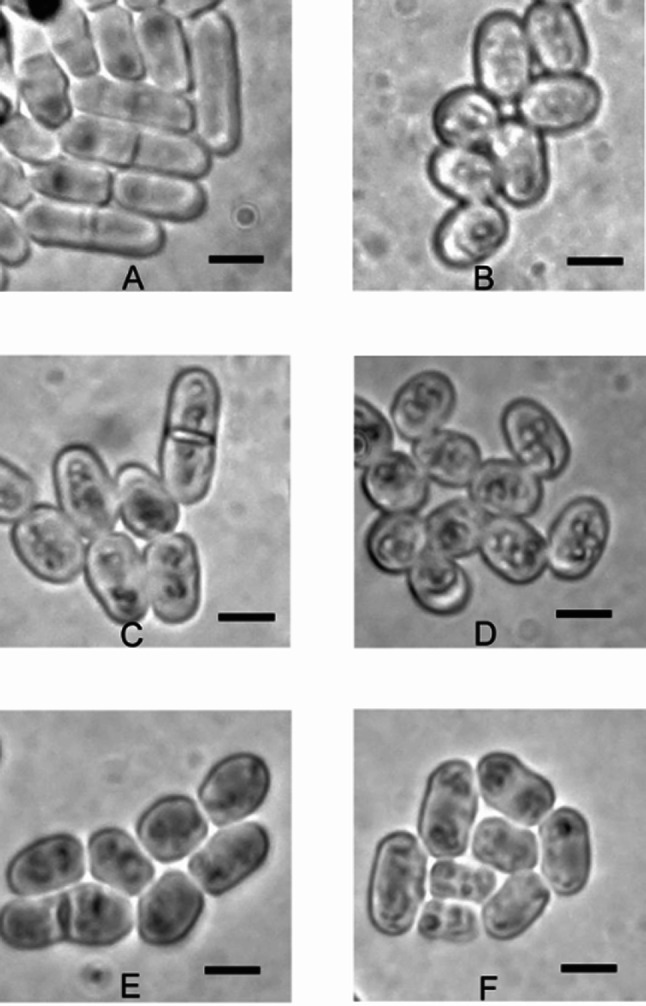

## Introduction

Actinomycetes are soil dwelling, filamentous bacteria which have a complex life-cycle, often comprising differentiation and sporulation. They are well known for their ability to synthesize a wide range of bioactive natural product compounds, many of which are of medical and commercial importance. For example, actinomycete bacteria produce a large number of polyene antifungal agents including nystatin [1], and candicidin/levorin [2] and the clinically used compound amphotericin B [3].

Although the problem of antimicrobial resistance in bacteria is well known, resistance in fungal pathogens is also becoming a significant clinical problem [4], with ~ 1.5 million disseminated or Invasive Fungal Infections (IFIs) requiring advanced treatment/hospitalization [5]. Most IFIs are caused by Candida [6, 7], Cryptococcus [8], Aspergillus [9–11] and Pneumocystis [5, 12–14] and are particularly prevalent in intensive care units, affecting elderly, and/or immunocompromised patients [12, 13]. Additionally, emerging fungal pathogens such as *Candida auris* and *C. glabrata*, present an increasingly serious clinical problem [14, 15].

Clinically used antifungal drugs, including the ergosterol synthesis inhibitors (*i.e*. the azoles, allylamines and morpholines), the polyenes (inhibition of ergosterol function), 5-fluorocytosine, (DNA synthesis inhibitor) and the echinocandins (β-glucan synthesis inhibitors) and griseofulvin (microtubule inhibitor) possess a range of limiting factors. These include fungal resistance (azoles, polyenes and 5-fluorocytosine), lack of broad-spectrum activity (echinocandins) and toxicity (polyenes). The most recently introduced class of new antifungal agents is the echinocandins (caspofungin) in 2001. There is thus a clinical need for improved antifungals, representing either new compound classes, or novel members of existing classes, which possess improved properties over existing drugs [16].

The fission yeast *Schizosaccharomyces pombe* is a rod-shaped unicellular eukaryote that grows by apical extension and divides by medial fission and septation [17]. It has a typical eukaryotic cell cycle and this together with the highly polarised growth pattern has made it an excellent organism for studying the underlying mechanisms involved in the processes of cell reproduction and the generation of cell form. In our recent study we described a novel screen for bioactive natural compounds produced by soil dwelling actinomycete bacteria using a microscopy-based approach to determine their effect on *S. pombe* cellular morphology, to identify chemical entities affecting these processes. [18]. As the mechanisms which determine cell morphology and cell cycle in prokaryotic and eukaryotic organisms are different, and as soil dwelling bacteria are competing in the same ecological niche as fungi, these eukaryotic-specific processes could be targets for bacterially synthesized secondary metabolites. We successfully identified a large number of actinomycete bacterial strains which produced bioactive compounds, which we classified according to their ability to induce eight major phenotypes in *S. pombe*. The success of this new screening approach has been validated by us identifying several known natural products (cycloheximide, streptonigrin, leptomycin) which induce *S. pombe* phenotypes which can be explained by their known modes of action.

To our knowledge this is the first time a screen based on the phenotypic effects of unknown compounds has been carried out using *Schizosaccharomyces pombe*. Gunji and co-workers determined the effects of various natural product antifungal compounds on the morphologies of several fungi, (including *S. pombe*), although the lack of comprehensive information provided by the study limits its usefulness [19]. The more recent study of Heisler describes adding 12 natural product compounds (though not medically used antifungals) to *S. pombe* and monitoring the phenotypes induced by imaging flow cytometry [20]. However, significantly, neither of these studies describes using the *S. pombe* phenotypic information as a guide to screening for unknown antifungal compounds.

The results of our screen indicated that it should be possible to use the *Schizosaccharomyces pombe* phenotypes induced by compounds as guides to identifying natural product/s, which specifically inhibit a particular cellular target, or molecular mechanism. To test this we designed a follow-up study to look at the *S. pombe* phenotypes induced by a number of known, commercially available antifungal agents. Once we had identified *S. pombe* phenotypes associated with known antifungals we would then focus on purifying the natural products from actinomycete strains capable of inducing such phenotypes identified during our previously reported screen. It was hoped the results would allow us to predict the cellular target and/or compound type of as yet unknown natural products identified in the screen, and so focus research on particular actinomycete strains producing classes of natural product which are of interest to us.

## Results and Discussion

**Fig. 1:**
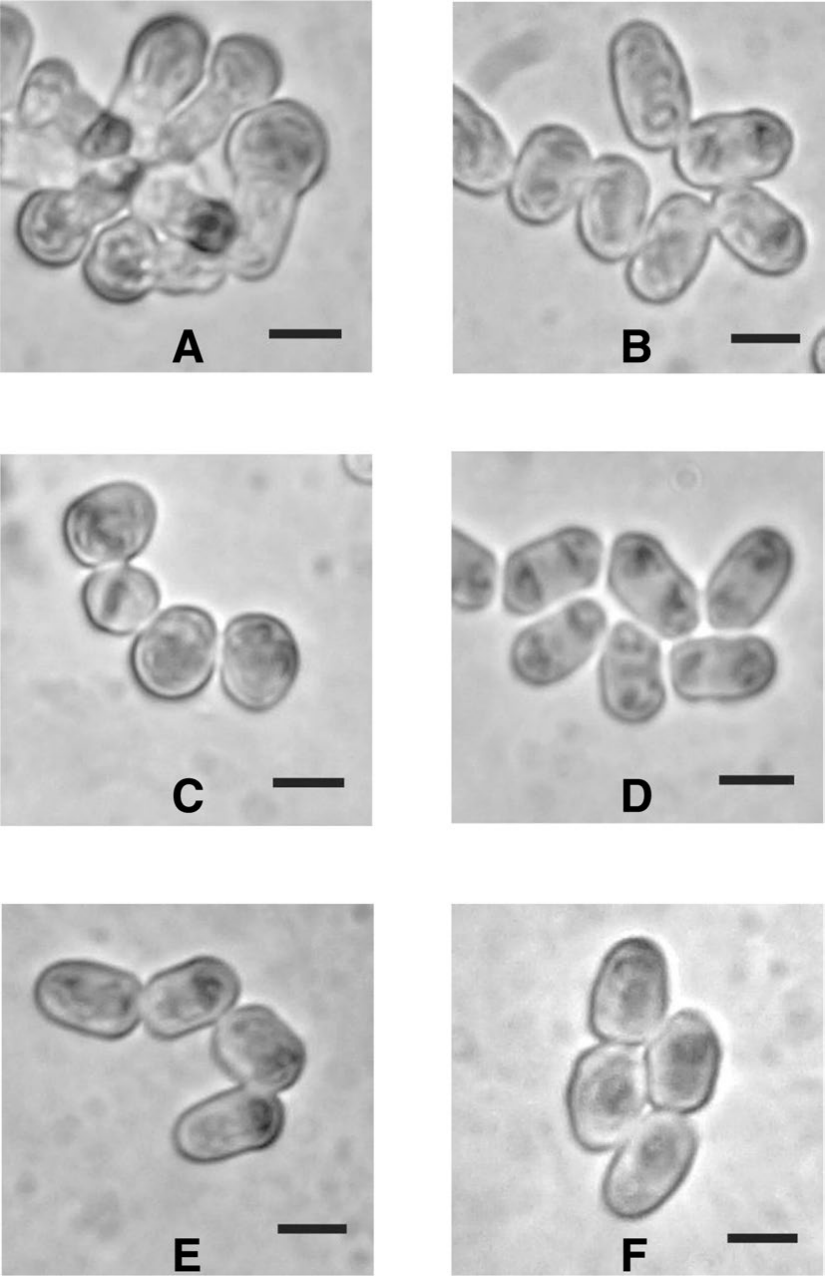
Examples of *Schizosaccharomyces pombe* cells from disc assays that exhibit different classes of cell shape defects in response to antifungal agents. **a** Caspofungin (2 mg/mL). **b** Streptothricin, (400 µg/mL). **c** Clotrimazole, (12.5 µg/mL). **d** Terbinafine, (0.195 µg/mL). **e** Amphotericin B, (315 µg/mL). **f** Nystatin, (800 µg/mL). Black bars represent 5 µm. Untreated *S. pombe* cells are illustrated in Fig. 2a

*Schizosaccharomyces pombe* bioassays were conducted on a series of commercially available antifungal compounds and these produced a variety of cellular/morphological phenotypes (Fig. 1). It is interesting to compare the large number (8–9 phenotypes) and striking nature of the *S. pombe* phenotypes which we observed during the screen of the *S. pombe* active natural products synthesised by the actinomycete strain collection [18] with the more limited number of phenotypes (2) observed herein.

**Fig. 2:**
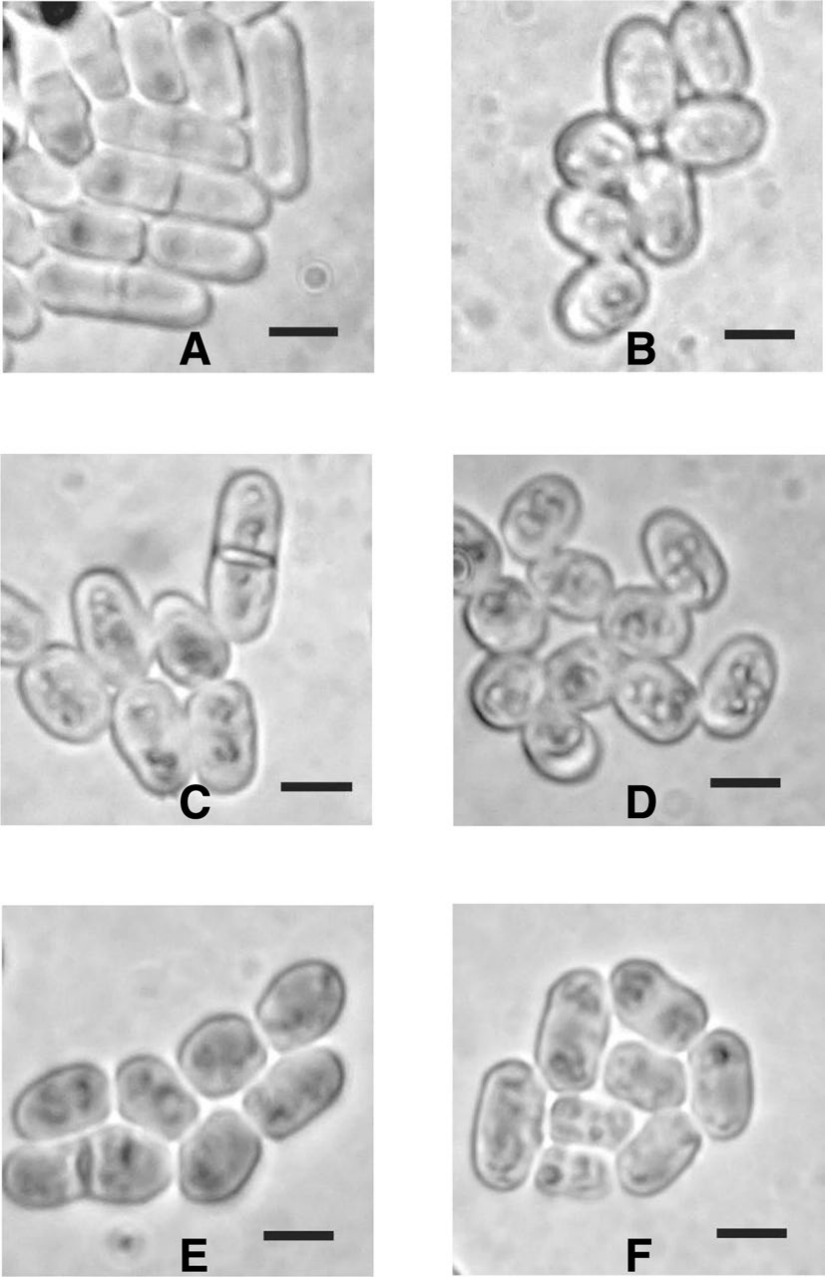
Examples of *Schizosaccharomyces pombe* cells that exhibit the rounded phenotype when challenged with actinomycete grown agar plugs. **a** SAK950 control, no actinomycete plug. **b** Strain E222. **c** Strain Wi37. **d** Strain DV7104. **e** Strain IS1. **f** Strain DV329. Black bars represent 5 µm

As expected, the β-glucan synthase inhibitor caspofungin induced an *Schizosaccharomyces pombe* phenotype, consistent with disruption of the cell wall, characterized by the presence of swollen, almost spherical cells (Fig. 1a). Although we did not observe this phenotype during the screen of the actinomycete strain collection it is reminiscent of the “cell wall” phenotype induced by the *Streptomyces griseus* strain 5307 (1B). This strain induced the phenotype due to its production of the protein synthesis inhibitor, cycloheximide, which leads to the build-up of unincorporated cell wall material at the cell termini, [18] probably due to the long-lived nature of β-glucan synthesis enzymes, which continue to synthesise β-glucan in the absence of other enzymes involved in its incorporation into the cell wall.

The remaining antifungal agents tested above induced a rounded *Schizosaccharomyces pombe* phenotype (Fig. 1b–f). This was one of the commoner phenotypes observed during our recent screen of actinomycete strains [18], in which we identified 57 strains that induced either the rounded (53), or broadly similar small (4), cell phenotypes. Amorolfine, terbinafine, fluconazole, ketoconazole, clotrimazole all inhibit the biosynthesis of ergosterol, whilst the polyenes amphotericin B, nystatin and filipin interfere with its functioning in the membrane. Thus, it is not surprising that they induce a similar phenotype in *S. pombe*, as they all act via the same target molecule. The SAK950 *S. pombe* strain possesses an *erg5Δ* mutation [21], and so is deficient is ergosterol [22], and although it was expected that SAK950 would therefore be resistant to polyenes [23], we found that its’ sensitivity to nystatin is almost identical to the wild-type strain 972. However, the SAK950 strain is markedly more sensitive than the wild-type to commercially available antifungal agents *e.g.* azoles (data not shown). Therefore, it seems that polyenes, in the absence of ergosterol itself, are still able to exert a killing effect, possibly by interacting with the ergosterol precursors present in SAK950.

Although we do not attempt a precise explanation of the “rounded” cell phenotype in terms of a precise mechanism of action of ergosterol targeting antifungals, sterols are well known to play an important role in maintaining the integrity of “lipid rafts” which are responsible, at least in part, for the correct localisation of protein complexes necessary for cell morphology/polarity [24–28]. Furthermore, we note that rounded phenotypes are consistent with reduced control of cell morphology/polarity, which leads to reduction, or loss, of the normal rod shape.

The bioassays indicated that the rounded phenotype was induced by many medically important antifungals. Therefore, we decided to investigate the actinomycete strains identified during the screen capable of inducing it in more detail (Fig. 2b–f). After re-examination of the 57 strains, we decided to focus on 46 of strains that grew in a reproducible manner and predictably induced a consistent *S. pombe* rounded phenotype.

**Fig. 3:**
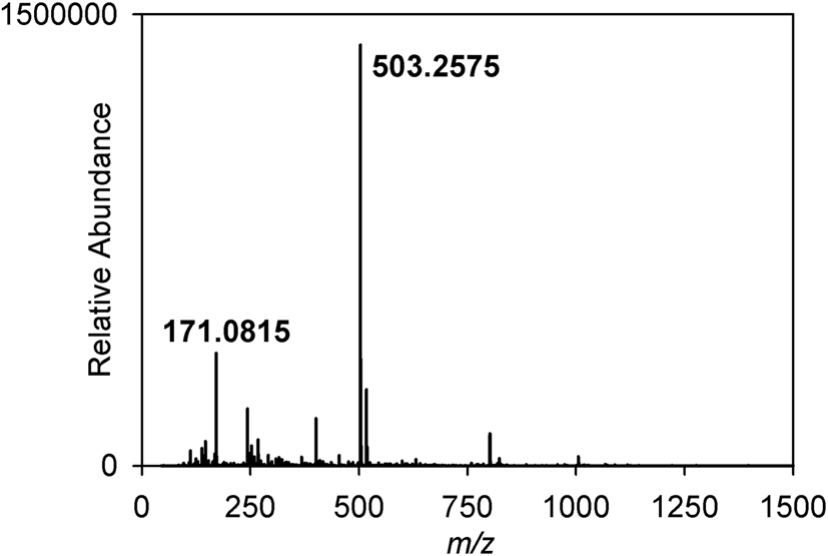
Mass spectrometry data for bioactive compound purified from E222. The *m/z* = 503.2575 [M+H]^+^ species represents streptothricin F whilst the *m/z* = 171.0815 [M+H]^+^ species represents a well-known streptothricin fragmentation product corresponding to the streptolidine moiety

When screening for bioactive natural products one of the more commonly found compounds is streptothricin [29], or derivatives thereof. These compounds, a group of nucleoside peptides comprising a carbamide-d-gulosamine core to which is attached a poly-β-lysine chain and the amino acid streptolydine, are thought to be inhibitors of protein synthesis and are toxic to both eukaryotes and prokaryotes. As the antifungal bioassays indicated that nourseothricin induces a “rounded” phenotype in *Schizosaccharomyces pombe* (Fig. 1b) it seemed likely that at least some of the actinomycete strains responsible for inducing this phenotype might synthesise streptothricins. To test this hypothesis we examined bioactivity against the *S. pombe* strain SAK950 and the *Escherichia coli* indicator strains DH5α pStrep^S^ (streptothricin sensitive) and DH5α pStrep^R^ (streptothricin resistant). Whilst the majority of the actinomycete strains had no effect on the growth of the DH5α pStrep^S^ strain, we found five strains capable of inhibiting its growth (Table S1). The fact that these strains did not inhibit the growth of DH5α pStrep^R^ indicated that the actinomycetes were producing streptothricin, or derivatives thereof, as opposed to some other compound possessing antibiotic activity that would not give rise to the differential inhibition of the streptothricin resistant and sensitive *E. coli* strains. *Prima facie*, these results strongly suggested that for these five strains the bioactive agents produced responsible for inducing the rounded phenotype were streptothricins. We decided to investigate one of these strains (E222) (Fig. 2b) in more detail to determine the form of the bioactive streptothricin molecule, as members of the streptothricin family can differ in the number of lysine moieties present [30, 31]. We purified the bioactive molecule (Fig. 3) using the protocol described above (see Materials & Methods, Purification of streptothricin). HPLC “Timeslicing” allowed correlation of the bioactivity *i.e.* induction of the “rounded” *S. pombe* phenotype, with a small peak eluting after 1.75 min which was collected and sent for mass spectrometry. The results (Fig. 3) indicated the presence of two species, the *m/z* = 503.2575 [M+H]^+^ species being readily identified using the “Dictionary of Natural Products” (http://dnp.chemnetbase.com/faces/chemical/ChemicalSearch.xhtml), as streptothricin F, the simplest form of streptothricin, comprising a single lysine moiety, whilst the *m/z* = 171.0815 [M+H]^+^ species was identifiable as a well-known streptothricin fragmentation product corresponding to the streptolidine moiety [32].

We do not attempt to interpret the “rounded” phenotype in terms of a precise cellular target of streptothricin as it is likely that the inhibition of protein synthesis induced by streptothricin has adverse effects on multiple cellular systems. Consistent with this, previous work has shown that rounded/ wide cells can be the result of a number of different cellular defects. For example, defects in nutrient monitoring, growth zone size and assembly, actin delocalisation or cell wall defects can all generate this phenotype [33–39].

Based on the results of the antifungal bioassays, which indicated that the polyene compounds amphotericin B and nystatin induce a rounded phenotype in *Schizosaccharomyces pombe* (Fig. 1e, f), it seemed likely that at least some of the rounded phenotype inducing actinomycete strains synthesise polyenes.

**Fig. 4:**
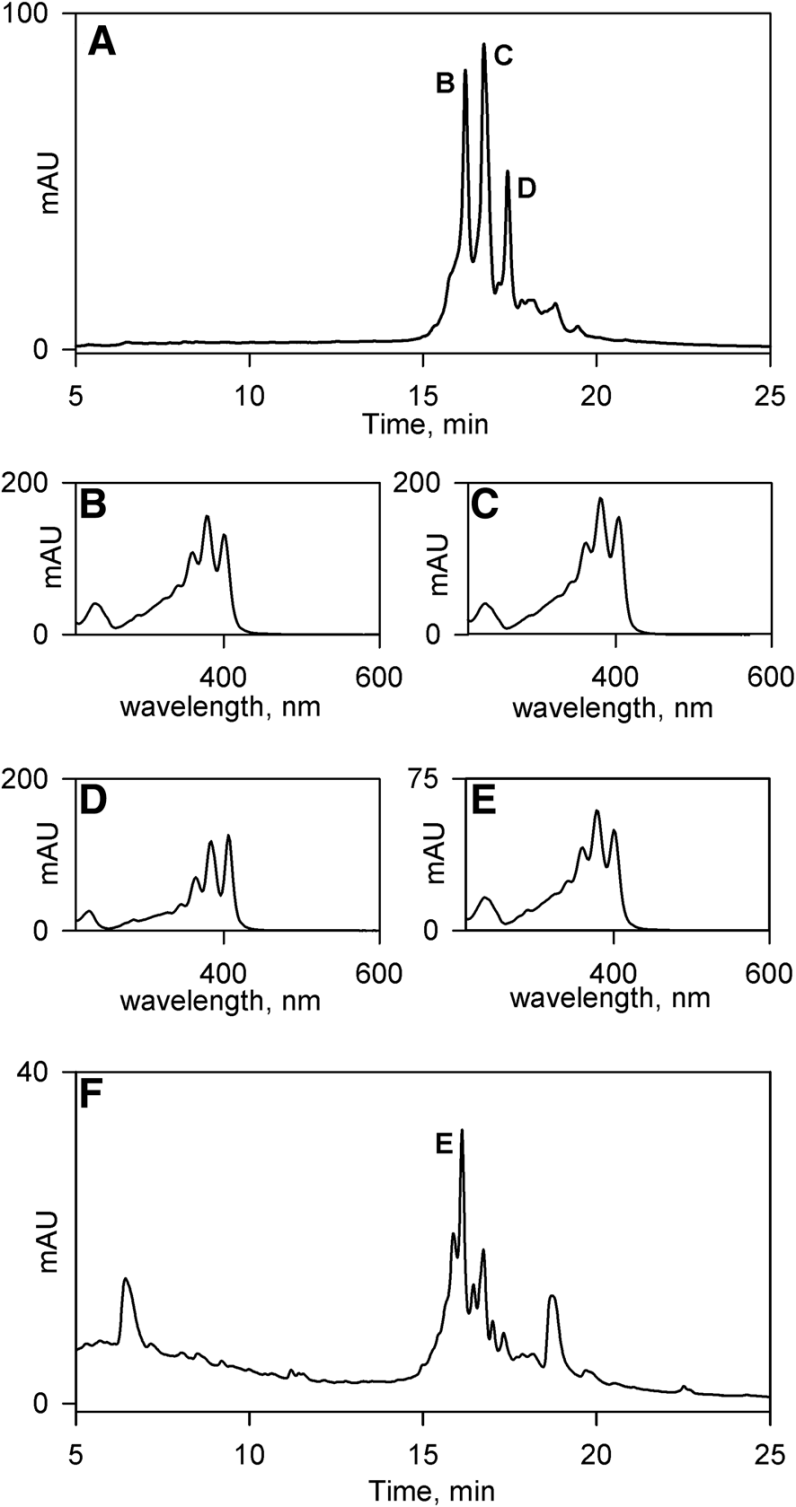
HPLC absorbance data (λ = 350 nm) for acetone derived extracts and associated polyene absorbance spectra. **a** HPLC absorbance data for strain YF989. **b** Absorbance spectrum for YF989 compound eluting at 16.2 min. **c** Absorbance spectrum for YF989 compound eluting at 16.7 min. **d** absorbance spectrum for YF989 compound eluting at 17.4 min. **e** Absorbance spectrum for DV7104 compound eluting at 16.2 min. **f** HPLC absorbance data for strain DV7104. The peaks in panels (**a**) & (**f**) which correspond to the compounds whose absorbance spectra are illustrated in panels (**b**)–(**e**) are labelled accordingly

Polyenes may readily be identified by their absorbance spectra which possess a characteristic triple-peak pattern in the λ = 300–400 nm region. Indeed, this “fingerprint” is frequently used to diagnose the presence of polyenes in natural product screens [40, 41]. We therefore developed a simple protocol for the rapid extraction of polyenes from crude cell derived material (see Experimental Procedures, Production of aqueous and acetone-based bacterial cell extracts) and applied this to all 46 strains in this study. This protocol was developed following the finding that for many of the strains the majority of the bioactivity was associated with the solid residue made by filtering freeze-thawed agar/cell slurry and that it could be released by extraction with acetone. Although trials with alternative solvents indicated that methanol was also effective, the use of less toxic acetone was preferred. The solvent derived extracts contained relatively few compounds when analysed by HPLC but importantly we were readily able to identify polyenes using the triple-peak absorbance spectrum as a diagnostic tool (Fig. 4b–e). Interestingly, we found that 26, over half of the strains, synthesized at least one polyene, and frequently a multiplicity of polyenes, forming a cluster of closely spaced peaks whose absorbance spectra strongly suggested they represented structurally related molecules (Fig. 4a). In addition to the polyenes within individual extracts being similar, the almost identical elution times of the polyenes in different extracts (Fig. 4a and f), together with their almost superimposable absorbance spectra (Fig. 4b and e), suggested that 24 strains, the majority of those synthesising polyenes, produced a single polyene species, or a closely related derivative.

**Fig. 5:**
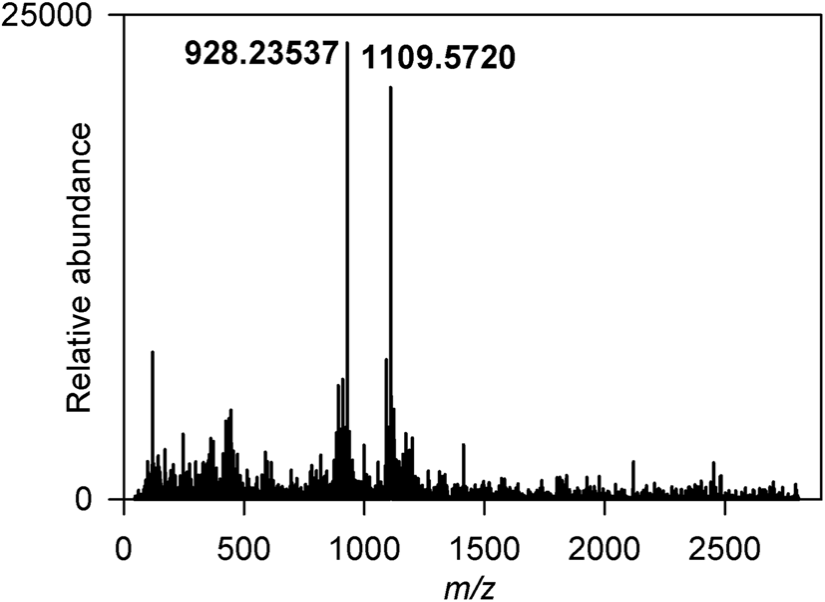
Mass spectrometry data for compound purified from DV7104. The *m/z* = 1109.5720 [M+H]^+^ species represents candicidin whilst the *m/z* = 928.23537 [M+H]^+^ species probably represents a well-known candicidin fragmentation product corresponding to candicidin – mycosamine - H_2_O

Strain DV7104 was selected for further analysis and the crude material produced by extracting freeze/thawed agar/cell slurry with acetone was concentrated and subjected to “flash” chromatography [see Experimental Procedures, Large-scale purification of polyenes (candicidin and filipin)]. The active fractions comprised several peaks and the largest of these was collected by “timeslicing” and analysed by mass spectrometry. The results (Fig. 5) indicated the presence of two species the *m/z* = 1109.5720 [M+H]^+^ species being readily identified as candicidin D (“levorin A2”) [3, 42–44], whilst the *m/z* = 928.2357 [M+H – mycosamine - H_2_O]^+^ species was identifiable as a well-known fragmentation product thereof [43]. We also note the similarity between the absorbance spectrum reported here and the published spectrum for candicidin [44, 45]. The fact that candicidin/levorin producing strains commonly synthesise a complex mixture of closely related active compounds also correlates with the multiple polyene molecules observed within each extract [42, 46]. To confirm the ubiquitous nature of candicidin D in our strains a second strain, Wi37, was analysed in a similar fashion to DV7104 and identical masses were obtained (data not shown).

**Fig. 6:**
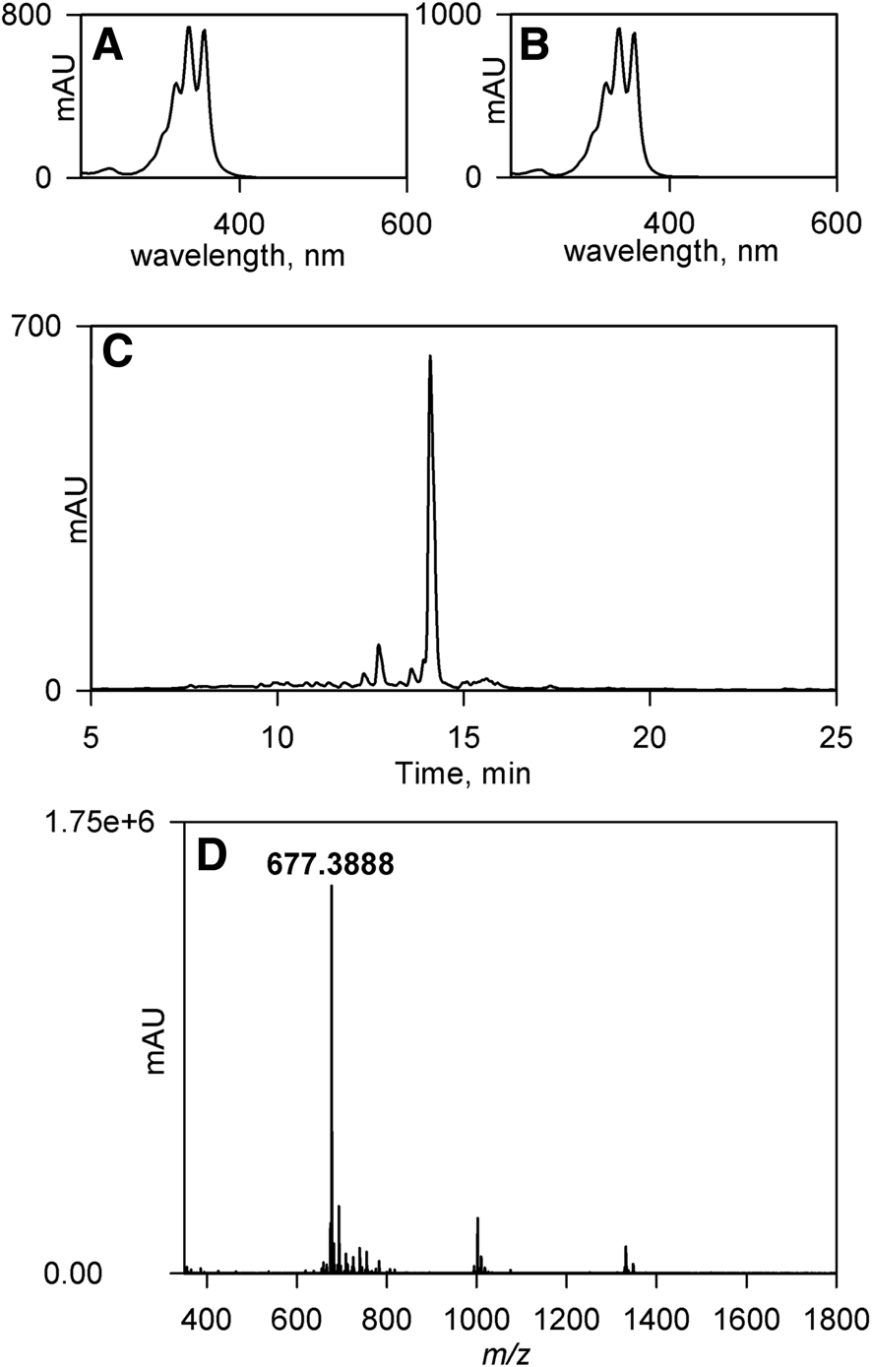
HPLC absorbance data (λ = 350 nm) for DV329 acetone derived extracts, associated polyene absorbance spectra and mass spectrometry data. **a** Absorbance spectrum for filipin. **b** Absorbance spectrum for DV329 compound eluting at 14.1 min. **c** HPLC absorbance data for strain DV329. **d** mass spectrometry data for purified compound from DV329 eluting at 14.1 min

Although the majority of the polyenes identified during the screen represent candicidin D, or variants thereof, two strains produced polyenes whose absorbance spectra differ from this. One of these is IS1, which we have previously described [18] and the other is DV329. The absorbance spectrum of DV329 has maxima at 324, 340 and 358 nm which is identical to that of filipin III (Fig. 6a, b). Accordingly, the crude extract produced from DV329 by extracting freeze/thawed agar/cell slurry with acetone was concentrated and subjected to “flash” chromatography [see Experimental Procedures, Large scale purification of polyenes (candicidin and filipin)]. Although the active fractions comprised several peaks (Fig. 6c), one was significantly larger than the others, and this was collected by “peak-picking” and analysed by mass spectrometry. The results (Fig. 6d) indicated the presence of a species at *m/z* = 677.3888 [M+Na]^+^, which allowed us to identify the molecule as filipin. We note that the absorbance spectrum of IS1 is also very similar to that of filipin and DV329 and this is consistent with our identification of this as fungichromin (also known as pentamycin, lagosin and cogomycin) following mass spectrometry of the purified molecule which gave a species at *m/z* = 693.379 [M+Na]^+^ [18].

We note that the literature contains numerous reports of screens for antifungal natural products which have yielded high proportions of polyene producing strains, many of which probably represent duplicates [41, 47–49]. However, we believe the dereplicated nature of the Goodfellow collection means the large number of polyene producing strains identified during the present study do not represent duplicates. Rather, it indicates both the widespread nature and frequent occurrence of polyene producing strains together with the use of an enrichment step for strains capable of inducing the *Schizosaccharomyces pombe* phenotype specifically associated with polyenes.

In a negative control experiment we additionally investigated 24 strains capable of inducing an elongated cell phenotype in *S. pombe* to determine the frequency of polyene production in a set of actinomycete strains not selected for their ability to induce the *Schizosaccharomyces pombe* “rounded” phenotype. The polyene extraction procedure was performed on samples of agar/cell material harvested when the strains were producing bioactive compound/s, as determined by induction of an elongated phenotype in the *S. pombe* plug bioassay. The results (not shown) indicated that only two of the 24 strains produced polyenes, identified via their absorbance spectra. The fact that the rounded phenotype was not observed in these two strains is probably due to the masking effect of the strong elongated phenotype which is frequently induced by production of DNA damaging compounds. These results suggest selecting strains on the basis of them inducing the rounded phenotype enriches for strains that produce polyenes, although the masking effect due to other phenotypes induced by production of additional bioactive compounds probably means that the number of polyene producers identified via the rounded phenotype in the Goodfellow collection understates the true figure.

**Fig. 7:**
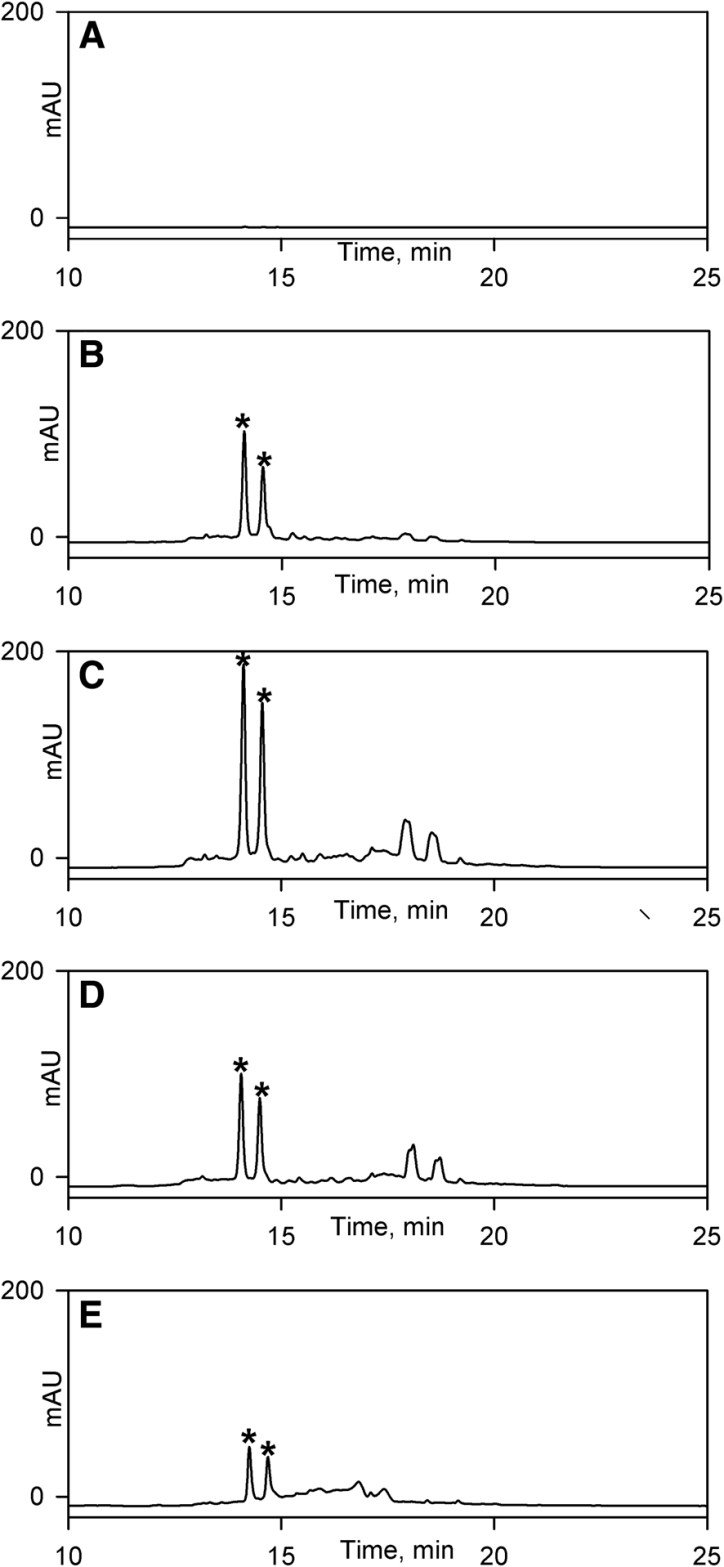
HPLC absorbance data (λ = 350 nm) for E222 acetone derived extracts from time-course experiment. **a** 24 h. **b** 36 h. **c** 48 h. **d** 60 h. **e** 72 h. The candicidin peaks are labelled with asterisks

**Fig. 8:**
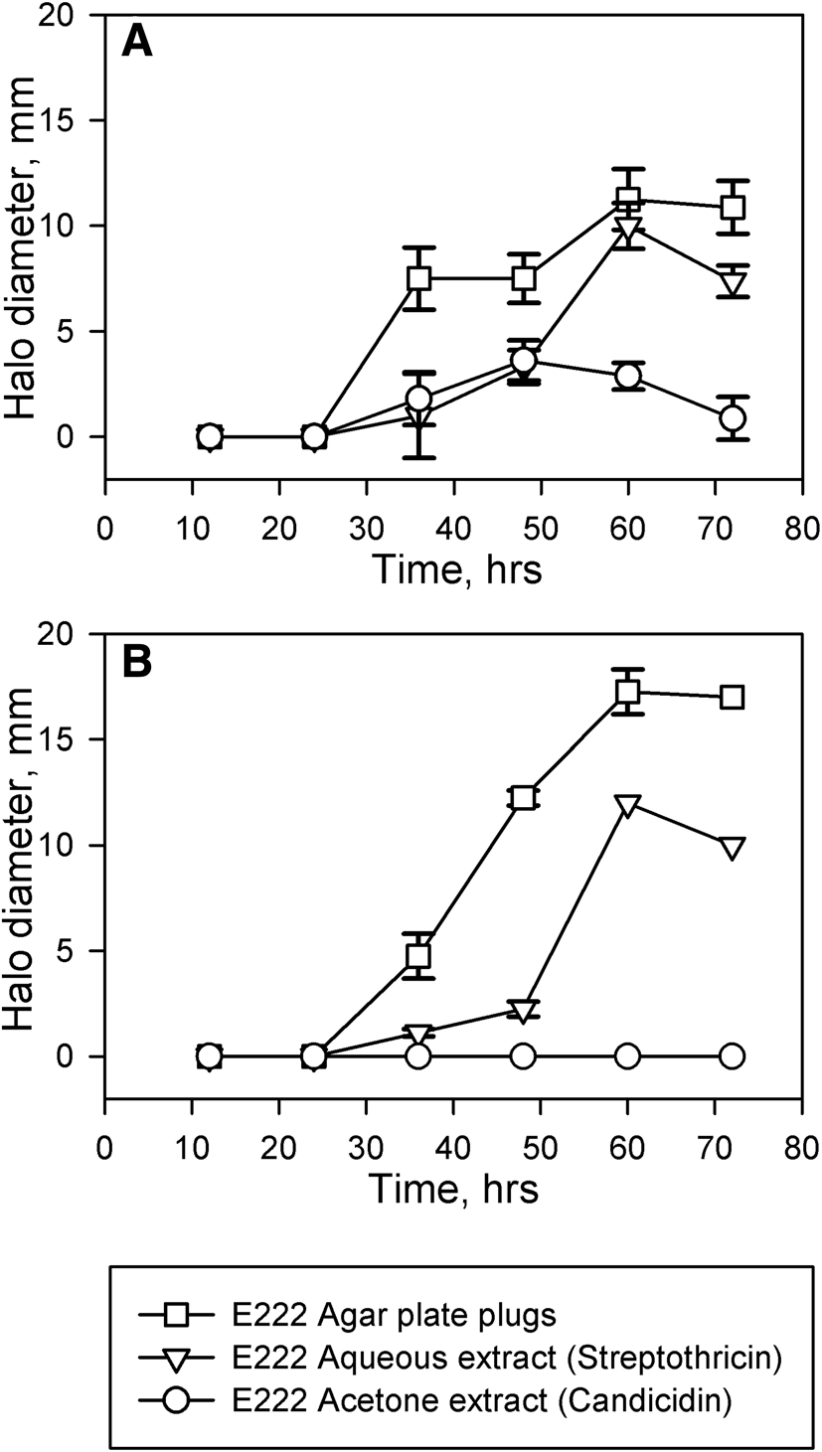
Zones of inhibition surrounding E222 samples from time-course experiment. **a** Halo diameters in *Schizosaccharomyces pombe* lawn. **b** halo diameters in DH5α pStrep^S^ lawn. Squares: agar plugs from lawn of E222; Triangles: filter paper disc treated with E222 aqueous extract (Streptothricin); Circles: filter paper disc treated with E222 acetone extract (Candicidin). Measurements are the halo diameters with the diameter of the plugs or filter paper discs subtracted

One of the more interesting results concerned the five strains already identified as producing streptothricin, which the results of the polyene assay show additionally produce candicidin D. An interesting question is whether the rounded *Schizosaccharomyces pombe* phenotype observed with these strains is due to candicidin or streptothricin. To resolve this issue we carried out in a time course experiment by correlating the onset of the rounded *S. pombe* phenotype in strain E222 [assessed by plug bioassay against *S. pombe* SAK950 (Fig. 8)] with the onset of biosynthesis of candicidin D [assessed using HPLC of acetone derived extracts of E222 (Fig. 7) and their disc bioassay activities against *S. pombe* SAK950 (Fig. 8)] and streptothricins [assessed by disc bioassay of aqueous extracts of E222 against *S. pombe* SAK950 and DH5α, pStrep^S^ (Fig. 8)]. The results (Figs. 7 and 8) indicated that biosynthesis of candicidin D was initiated slightly earlier than that of streptothricin and candicidin D production peaks at 48 h whilst streptothricin production peaks at 60 h.

The induction of the rounded phenotype correlated with the onset of candicidin D biosynthesis, but before streptothicin was detectable. This suggests that candicidin D is mainly responsible, at least initially (24–36 h), for inducing the rounded phenotype but that streptothricin plays a larger role in inducing the phenotype in the later stages of the time course (48–72 h). However, a blind purification of the bioactive compound from E222 was carried out and only streptothricin was purified (Fig. 3), suggesting that this molecule is probably the major contributor to the phenotype, at least under the culture conditions used. This result is broadly consistent with the results of the time course, in which the haloes due to streptothricin in the aqueous extract were significantly larger than the haloes due to candicidin D present in the acetone-based extract. However, we cannot exclude the possibility that differences in efficiency of the extraction procedures and/or the stability of the natural product compounds may also partly account for the differences between the two compounds.

Polyene biosynthetic gene clusters are commonly regulated by PAS-LuxR transcriptional regulators [50] and these have been shown to exhibit cross-talk with other secondary metabolite gene clusters and coordinate biosynthesis of polyenes and other natural products. For example, the polyene candicidin and the antimycins—hybrid non-ribosomal peptide/polyketide molecules, have been shown to be co-regulated [51], as have the polyene filipin and the polyketide oligomycin [52]. Heterologous expression of the LuxR family positive activator *pimM* gene from the pimaricin (polyene) gene cluster has also been shown to activate streptothricin production, in addition to biosynthesis of a heptaene polyene in *Streptomyces albus* [53]. The largely overlapping production of candicidin D and streptothricin by E222 observed here is broadly consistent with coordinate regulation of a polyene and another biosynthetic gene cluster by a polyene cluster located transcriptional regulator. Although the reason for the co-regulation/production of candicidin and other natural products is unknown, synergistic killing effects of a polyene, amphotericin B, and other natural product compounds on fungi have been observed previously. For example, amphotericin B and tetracycline against *Candida albicans* [54], amphotericin B and azithromycin against *Fusarium* [55], amphotericin B and rifampicin against *C. albicans* [56], amphotericin B and doxycycline against *Candida *spp.[57]. It has been postulated that these effects are due to amphotericin B damaging the fungal cell membrane, which may facilitate the entrance of the other bioactive compound into the cell [55], and this synergistic relationship may explain the co-regulation/production of candicidin and other natural products by actinomycetes.

The fact that a large proportion of the strains used in the study produced the same natural product (*i.e*. candicidin, and to a lesser extent streptothricin) prompted a taxonomic/phylogenetic follow-up study of the strains. This was designed to investigate whether this phenomenon was due to a large number of duplicated, closely related strains or a large number of unrelated strains coincidentally producing identical bioactive compounds. For example, candicidin is known to be produced by a number of different Streptomyces species including *Streptomyces griseus* [3], *S. hygroscopicus *var *jinggangensis* [58], *S. acrimycini* [59], *S. helvoloviolaceus* [60] *S. fradiae* [61] and *S. levoris* [62]. The 16 s rRNA gene was sequenced for all of the strains and the data (Table S1) used to assign the strains to their closest defined species and to construct a phylogenetic tree (Fig. S1).

The results indicate that the candicidin only producing strains do form a distinct cluster (Fig. S2) with many strains being identified as, or closely related to, *Streptomyces hydrogenans* and *S. albidoflavus*, with a single candicidin producing strain (M017) being identified as *S. enissocaesilis*. As the candicidin/streptothricin producing strains also form a cluster (Fig. S3) with the strains being identified as, or closely related to, *S. enissocaesilis*, it is tempting to interpret this in terms of M017 having either lost the ability to produce streptothricin, or never acquired it. Interestingly, M017 indicates that candicidin production in a *S. enissocaesilis* strain is sufficient to induce the rounded/small *Schizosaccharomyces pombe* phenotype, despite the apparently major role played by streptothricin, as the abovementioned results indicate (Figs. 7 and 8).

*Streptomyces albidoflavus* has previously been shown to produce candicidins [63] whilst *Streptomyces hydrogenans* has been previously reported to produce an antifungal agent [64], although this was later shown not be a polyene [65], and *S. enissocaesilis* has not been reported to produce antifungal agents.

The two pentaene polyene producing strains, IS1 and DV329, related to *Streptomyces misionensis* (fungichromin) and *S. spiralis* (filipin), respectively, do not form a discrete cluster, and neither species has been previously reported to produce antifungals, although the production of filipin by *S. filipensis* [66], *S.* *miharensis* [67] and *S.* *avermitilis* [68] and the production of fungichromin by *S. rochei* [69], *S. albogriseolus* [70], *S. padanus* [71] and *S. cellulosae* [72] are well known.

The 20 non-polyene producing strains are more heterogeneous in nature, comprising three *Micromonospora* strains in addition to *Streptomyces* species. Some of these species are represented several times *i.e*. *S. niveus*, *S. kunmingensis* and *S. camponoticapitis*, (5, 3 and 2 times respectively). However, as the 16 s rRNA gene sequence data indicates these strains are not true duplicates in that they possess different numbers of mismatches relative to the type species and probably represent similar strains isolated from different locations at different times, rather than multiple versions of the same strain isolated from the same location at the same time.

To extend the study we decided to investigate whether strains in the Goodfellow collection not previously identified as inducing the “rounded” or “small” *Schizosaccharomyces pombe* phenotypes, but which on the basis of 16 s rRNA data were identified with, or closely related to, strains identified in the course of the present study produced natural products capable of inducing these phenotypes. We therefore selected eight strains related to the candicidin only producers (MU1476, MU147-B, MU1858, MR620, A62, S34, WAB680 & MDA8-444) whose closest type strains were *S. hydrogenans* and *S. albidoflavus* (Table S2 & Fig. S2). We also selected three strains related to the streptothricin producers (E223, 5231 & B12) for further study whose type strains were closest in the tree to *S. enissocaesilis* (Table S2 & Fig. S3). All strains were each streaked out on GYM plates on successive days for 7 days and bioactivity against *S. pombe* and the presence of a rounded or small phenotype assessed using plug assays. In the case of suspected streptothricin producers (selected for their relatedness to *S. enissocaesilis*) plugs from plates shown to be capable of inducing the rounded or small *S. pombe* phenotypes were tested against DH5α pStrep^R^ and pStrep^S^. Additionally, acetone-based extracts were produced from plates capable of inducing the rounded or small *S. pombe* phenotypes and assayed for the presence of polyenes by HPLC. The results indicated that all of the strains selected were capable of inducing the rounded or small *S. pombe* phenotypes. In addition to the two positive control strains, Wi37 and DV7104, all of the strains selected for their relatedness to *S. hydrogenans* or *S.* *albidoflavus* produced candicidin, as did all three strains selected for their relatedness to *S. enissocaesilis*. However, it is interesting that in addition to the positive control strain (E222) one strain close in the phylogenetic tree to *S. enissocaesilis, i.e*. *S. plicatus* (E223), produced streptothricin, whereas two others, *i.e*. *S. rochei* [5231 T (12)] and *S. mutabilis* (B12), did not. The fact that B12 produced haloes on both DH5α pStrep^S^ and pStrep^R^ strain (data not shown) indicated that it synthesized at least one non-streptothricin antibiotic.

There have been relatively few previous reports investigating the relationship between actinomycete speciation and natural products. Some species have been shown to be very homogeneous with respect to their complement of natural product biosynthetic gene clusters *e.g. Streptomyces diastaticus subsp ardesiacus* [73] and *S. pratensis* [74], whilst others display a more heterogeneous repertoire, *e.g. S. albus* [75], *Nocardia brasiliensis* [76], and *Salinispora pacifica* [77]. Although of limited scope, the follow-on study suggests that strains related to *S. enissocaesilis* are heterogeneous with respect to streptothricin production and homogeneous with regard to candicidin biosynthesis.

## Conclusion

We have designed here a screen using a microscopy-based approach to determine the effect of known antifungal agents on *Schizosaccharomyces pombe* cellular morphology and used these diagnostic phenotypes as a basis for the identification of bioactive natural compounds produced by soil dwelling actinomycete bacteria. We found the rounded or small phenotypes were frequently induced by clinically used antifungals and our attention was thus focussed on actinomycete strains capable of inducing similar effects and potentially synthesizing compounds of similar utility.

This approach falls within the scope of the modern neoclassical phenotypic drug discovery (PDD) movement where physiology-based approaches, as opposed to a molecular targeted drug discovery (TDD) approach, are combined with technological advances [reviewed: [78]]. Prior art screening techniques have either used simple, live/dead screens where candidate molecules may affect a range of targets, or focussed phenotypic screens looking for inhibition of a single target/molecular mechanism. In contrast our screen combines both approaches *i.e.* the breadth of a simple screen (as we are looking for inhibition of a range of targets) combined with some of the specificity of a targeted phenotypic method (as the *S. pombe* phenotype may be indicative of a limited number of molecular targets or modes of action).

The results confirm the validity of the approach and, following the discovery that polyene compounds and streptothricin both induce a rounded phenotype, we were able to show that many of the actinomycete strains capable of inducing similar phenotypes also produced the same compounds. In particular the ‘rounded strains’ were enriched for those synthesizing one particular polyene, candicidin, which was synthesized by over half of the strains, although the fact that two other polyene (filipin and fungichromin) producing strains were also identified indicates that our assay is not only identifying candicidin.

We went on to show by taxonomic analyses of the 16 s rRNA gene sequences that the majority of the candicidin producing strains were closely related to *Streptomyces hydrogenans* or *S. albidoflavus*, whilst strains that are related to *S. enissocaesilis* ubiquitously produced candicidin, and frequently streptothricin The results of a follow-on study investigating whether hitherto untested strains related to these species also induce the “rounded” or “small” *S. pombe* phenotypes, and produce similar natural products, indicated that this was indeed the case, with strains closely related to *S. hydrogenans* or *S.* *albidoflavus* producing candicidin, whilst those related to *S. enissocaesilis* produced candicidin and sometimes streptothricin. The results suggest that taxonomic data may be of value, particularly when searching for a strain in which a biosynthetic gene cluster (BGC) of interest is active, as BGC’s are cryptic in 77% of cases, so one may need to screen four related isolates to find a strain in which it is active [79].

It has been noted that “*future (drug) discovery efforts would benefit greatly from a focus on gifted phylogenetic groups coupled with prescreening to reduce the number of close relatives*” [79]. This description aptly describes the extensively dereplicated Goodfellow collection, which represents a rich taxonomic resource that, when correlated with the literature relating to natural product biosynthesis from particular species, may prove to be a powerful tool in identifying species producing novel bioactive compounds. This approach is likely to be especially effective when combined with the results of the *Schizosaccharomyces pombe* phenotypic assay which, as the results presented here demonstrate, can highlight strains likely to produce particular classes of antifungal agents of interest.

## Experimental Procedures

### Yeast Strains, Growth Conditions and Media

The *Schizosaccharomyces pombe* SAK950 strain (h+ *ade6-M216, leu1-32, ura4-D18, caf5::bsdR, pap1Δ, pmd1Δ, mfs1Δ, bfr1Δ, dnf2Δ, erg5::ura4*+) [21] used in this study was grown at 30 °C in YE4S [80].

### Plug Assay

1 ml pipette tips were used to cut agar plugs from the lawns of bacterial strains on GYM agar (see below). Bacterial plugs, plus an agar only control plug were placed on a YE4S plate seeded with 5 × 10^5^ mid log phase SAK950 cells, or on Nutrient Agar (Oxoid™) plates seeded with either 5 × 10^8^ early stationary phase *E. coli* DH5α pStrep^S^ or DH5α pStrep^R^ (see “Construction of a streptothricin resistance cassette” below). This procedure was typically carried out after 3, 5, 7 and 9 days of bacterial growth. Plates were incubated overnight at 30 °C and screened the following day for the presence/absence of a halo in the indicator organism lawns surrounding the plugs and, in the case of SAK950, microscopically for the morphological appearance of the *S. pombe* cells. As biomolecules diffuse through the agar at different rates and have effects at different concentrations, the phenotype of the cells was examined from the plug to the edge of the halo.

### Filter Paper Assay

Filter papers soaked in extract (10–30 µL) (Whatman Cat No: 2017-006) or appropriate dilutions of DMSO/H_2_O as controls, were used, instead of bacterial plugs, as described above. For the assays of known, commercially available antifungal agents stock solutions of the compounds including the polyenes, amphotericin B (Fisher Scientific), nystatin and filipin (Cambridge Bioscience); the allylamines, amorolfine (LKT) and terbinafine (Sigma); the azoles, clotrimazole (Melford Laboratories), fluconazole (Melford Laboratories), and ketoconazole (Melford Laboratories); the echinocandin, caspofungin (Sigma) and the protein synthesis inhibitor streptothricin (Santa Cruz), were prepared in DMSO or water and diluted in water to generate the serial dilutions described in Table 1 and 10 µL of each dilution was used.

**Table 1:** Commercially available antifungal agents used in the "Filter paper assay" and details of how the stock solutions were prepared and diluted

Antifungal agent	Stock concentration and solvent	Concentration of diluted antifungal agents							
Caspofungin	20 mg/mL DMSO	2 mg/mL	1 mg/mL	800 µg/mL	600 µg/mL	400 µg/mL	200 µg/mL		
Streptothricin	2 mg/mL H_2_O	400 µg/µl	200 µg/µl	100 µg/µl	50 µg/µl	25 µg/µl	12.5 µg/µl	6.25 µg/µl	
Clotrimazole	3 mg/mL DMSO	100 µg/mL	50 µg/mL	25 µg/mL	12.5 µg/mL	6.25 µg/mL	3.125 µg/mL	1.56 µg/mL	0.78 µg/mL
Terbinafine	10 mg/mL DMSO	25 µg/mL	12.5 µg/mL	6.25 µg/mL	3.125 µg/mL	1.56 µg/mL	0.78 µg/mL	0.39 µg/mL	0.195 µg/mL
Amphotericin B	40 µg/mL DMSO	10 mg/mL	5 mg/mL	2.5 mg/mL	1.25 mg/mL	0.63 mg/mL	0.32 mg/mL	0.16 mg/mL	
Nystatin	5 mg/mL DMSO	1 mg/mL	800 µg/mL	600 µg/mL	400 µg/mL	200 µg/mL	100 µg/mL		
Filipin	5 mg/mL DMSO	5 mg/mL	4 mg/mL	2 mg/mL	1 mg/mL	500 µg/mL	250 µg/mL	125 µg/mL	
Amorolfine	1 mg/mL DMSO	2 µg/mL	1 µg/mL	0.5 µg/mL	0.25 µg/mL	0.125 µg/mL	0.0625 µg/mL	0.03125 µg/mL	
Ketoconazole	2 mg/mL DMSO	200 µg/mL	100 µg/mL	50 µg/mL	25 µg/mL	12.5 µg/mL	6.25 µg/mL	3.125 µg/mL	1.56 µg/mL
Fluconazole	25 mg/mL DMSO	2.5 mg/mL	1 mg/mL	500 µg/mL	250 µg/mL	125 µg/mL	62.5 µg/mL	31.25 µg/mL	

### Microscopy

Bright field images of live cells were acquired on a Zeiss Axioskop 40 microscope with a Zeiss 63X/1.4 oil ph3 objective and a Zeiss Axiocam MRm camera. For the plug assay plates cell images were captured with a Sony HD AVCHD camera using a Zeiss Akioskop 40 microscope with a X50/0.56 Nikon objective and 2.5X optivar.

### Bacterial Strains, Growth Conditions and Media

The Demuris bacterial strain collection comprises over 10000 strains, collected over 40 years of research into actinomycete taxonomy by Professor M. Goodfellow of The University of Newcastle UK, together with a collection of marine derived actinomycetes. It is notable for the diversity of the strains, which in many cases were obtained from ecologically unexplored habitats, or by novel isolation methodologies (or both), and the degree to which the collection has been de-replicated so as to remove multiple identical strains.

The study focussed on 46 strains previously shown to inhibit the growth of *S. pombe* and induce either the “rounded” phenotype or similar “small” phenotype and are listed in Supplementary Table S1 [18], with one additional strain (AUS7002). These strains were resuscitated from storage at − 80 °C on Oatmeal Agar plates as described previously, and for analysis confluent bacterial lawns were streaked from the resuscitation plates on GYM agar plates and grown at 30 °C [18]. All actinomycete strains were streaked onto GYM agar plates and grown for varying lengths of time typically 3, 5, 7 or 9 days until they strongly produced natural product compounds, as determined by assessing bioactivity (halo size and phenotype induced in *S. pombe*) using the “plug assay” (see above).

For standardized growth of strain E222 to investigate the timing of onset of production of candicidin and streptothricin GYM plates were inoculated with sufficient titred spores to give ~ 2500 colonies per plate. Spores were prepared as described previously [81].

### Production of Aqueous and Acetone Based Bacterial Cell Extracts

Actinomycete strains were streaked onto GYM agar plates and grown until they strongly produced natural product compounds, as determined by assessing bioactivity using the “plug assay” as described in “Bacterial strains, growth conditions and media”. Agar from three plates per strain was harvested, disrupted by passaging through a 50 ml syringe and frozen overnight at − 20 °C. The semi-dried agar/mycelial derived material produced by squeezing the thawed slurry through muslin was resuspended in a volume of acetone equivalent to the volume of aqueous extract produced therefrom and incubated overnight at 4 °C. Acetone based extract was produced by squeezing the mixture through muslin, followed by filtering it through Whatman filter paper. Polyenes were frequently found to be present in the acetone-based extract, as opposed to the aqueous extract and this was commonly assayed by HPLC to assess their production.

#### Purification of Streptothricin

For the purification of streptothricin aqueous extracts produced by squeezing freeze/thawed agar/cell slurry, generated by growth of E222 on Medium I plates [82], through muslin had washed Amberlite™ XAD16N resin beads (Sigma) added (20 g l^−1^) to adsorb bioactive compounds. The compounds were released by washing the resin with a volume of methanol equal to the volume of the original aqueous extract and the methanol was removed by rotary evaporation (Büchi) to obtain a water-based concentrate. The concentrate, after filtering through Whatman filter paper and the addition of methanol to 10%, was passed through a pre-packed “reverse phase” C18 25 g SNAP “flash” chromatography column (Biotage) and the bound compounds eluted in a 90–0% water—methanol gradient using a Biotage “Isolera One” chromatography machine. Fractions containing bioactive compounds were identified by spotting 30 µL aliquots onto filter paper discs, see above (filter paper assay). Active fractions producing a halo of cells with the expected characteristic phenotypes were pooled and the methanol removed by a GeneVac Series II system equipped with a GeneVac VC3000TA condenser unit. Material from active fractions was analysed/further purified by HPLC.

### *Large Scale Purification of Polyenes *(*Candicidin and Filipin*)

The actinomycete strains were streaked onto GYM agar plates and assayed as described in “Bacterial strains, growth conditions and media”. Plates (~ 200) made using 5 L of agar per strain were harvested, the agar/mycelial mass disrupted by passaging through a 50 mL syringe and frozen overnight at − 20 °C. The semi-dried agar/mycelial derived material produced by squeezing the thawed slurry through muslin was resuspended in a volume of acetone equivalent to the volume of discarded aqueous extract and incubated overnight at 4 °C. Acetone based extract was produced by squeezing the mixture through muslin, followed by filtering it through Whatman filter paper. The acetone was removed by rotary evaporation (Büchi) to obtain a water-based concentrate which then had sufficient water added so as to make it up to the original volume of discarded aqueous extract (~ 500 mL). After filtering and addition of methanol to 10%, the extract was passed through a pre-packed “reverse phase” C18 Ultra 12 g “flash” chromatography column (Biotage) and the bound compounds eluted in a 90–0% water—methanol gradient using a Biotage “Isolera One” chromatography machine. Fractions containing bioactive compounds were identified by spotting 30 µL aliquots onto filter paper discs, which were used in the “filter paper assay”. Active fractions producing a halo of cells with the expected characteristic phenotypes were pooled and the methanol removed by a GeneVac Series II system equipped with a GeneVac VC3000TA condenser unit. Material from active fractions was analysed/further purified by HPLC.

### HPLC

All analyses/purifications were conducted using an Agilent Technologies 1260 Infinity liquid chromatography machine equipped with either an Agilent Zorbax SB-C18 5 µM 4.6 × 150 mm column, a Waters Symmetry™ C18 4.6 × 250 mm column or an Agilent Eclipse Plus C18 3.5 µM 4.6 × 150 mm column equipped with a Hichrom C18 guard column.

Streptothricin derived from E222 was eluted using a water—acetonitrile gradient over 30 min (0–100% acetonitrile), with a flow rate of 1 mL min^−1^ and monitoring with a DAD array at λ = 210, 254, 273, 280, 300, 450 and 600 nm relative to λ = 360, and λ = 350 nm relative to λ = 500 nm. Polyenes were eluted using a water—acetonitrile gradient over 30 min (20–100% acetonitrile), with a flow rate of 1 mL min^−1^ and monitoring with a DAD array at λ = 254, 270, 300, 400, 450, 500 & 600 nm relative to λ = 360, and λ = 350 nm relative to λ = 500 nm. For all compounds fractions (0.5 mL) were collected in a 96 well block and active fractions identified by the filter paper assay. Following correlation of peaks in the absorbance traces with the active fractions the method was repeated using an Agilent 1260 integrated fraction collector so as to “time-slice” and collect column eluate corresponding to the bioactive eluate into glass tubes.

### Mass Spectrometry

After removal of solvent using the GeneVac Series II and addition of 25% methanol to enhance solubility the polyene samples (candicidin, filipin) were analysed by electrospray mass spectrometry (ESI-MS) using an LTQ-FT (Thermo) mass spectrometer with a 7 T magnet at the Pinnacle Laboratory (University of Newcastle). Experiments were run with a parent/precursor scan at 100,000 resolution. MS/MS fragmentations were carried out in the ion-trap (LTQ) stage of the instrument. Streptothricin and candicidin were analyzed on an LC-MS (Bruker micrOTOF, Agilent 1260 HPLC system, Zorbax Eclipse Plus column (3.5 μm 100 × 4.6 mm).

### Construction of a Streptothricin Resistance Cassette

The streptothricin acetyltransferase gene, *sat* DNA sequence (GeneID:1238709, ProteinID: NP065310.1 from the *E. coli* K-12 plasmid R721 (RefSeq:002525.1), was synthesised de novo (DC Biosciences) with an *NdeI* restriction site overlapping the starting ATG codon and a *BamHI* restriction site after the TAA stop codon. The *sat* gene was cloned into *NdeI/BamHI* cut pUC57 in-frame with the *lac* promoter to generate pStrep^R^. The negative control plasmid, pStrep^S^, was generated by *StuI/Eco53kI* digestion of pStrep^R^ followed by self-ligation of the plasmid backbone. The activity of both these constructs in *E. coli* DH5α were tested and pStrep^R^ shown to provide resistance against streptothricin from both leaky and IPTG-induced expression from the *lac* promoter (data not shown), whilst pStrep^S^ did not confer resistance to streptothricin.

### Genomic DNA Extraction and rRNA Gene Sequencing

All actinomycete strains were streaked onto Modified Bennetts Medium agar plates and grown for varying lengths of time typically 3–5 days and then pea sized amounts of mycelium were harvested using sterile loops and placed in 1.5 mL tubes containing 0.3 g of acid-washed sterile glass beads (106 µm, Sigma), 400 µL TE buffer and 200 µL of phenol/chloroform/ isoamyl alcohol. The mycelium was disrupted using a FastPrep™ FP120 (BIO 101 Thermo Savant) machine (4.5 m/s for 45 s with cooling on ice for 2 min in between). The disrupted mycelium was centrifuged (13000× *g*) for 5 min and the supernatant (200 µl) transferred to a new tube and 20 µL of RNase (1 mg/mL, Sigma) added and incubated for 2 min at room temperature, followed by addition of 20 µl Proteinase K (50 mg/mL, Fisher). After incubation at 55 °C for 15 min, 20 µl of 5 M NaCl and 500 µL of ice-cold ethanol were added and the tube mixed by inversion and incubated for 1 h at − 80 °C before being centrifuged at 6000× *g* for 2 min. The pellet was washed in 70% ethanol and then dried for 1 h at 37 °C then re-dissolved in 100 µL of water and the presence of DNA verified using agarose gel electrophoresis.

The rRNA gene was amplified using the BIOTAQ™ DNA Polymerase kit (BioLine) in accordance with the manufacturers’ instructions. The primers “27F” (5′AGAGTTTGATCMTGGCTCAG3′) and “1525R” (5′AAGGAGGTGWTCCARCC3′). The reaction mix comprised 5 µL of 10X NH_4_ Reaction Buffer, 3 µL of 50 mM MgCl_2_ Solution, 0.5 µL of 100 mM dNTP mix, 0.2 µM of each primer, 1 µL (~ 50 ng) of genomic DNA template (concentration determined using a Nanodrop™ ND-1000 spectrophotometer), 1 µL BIOTAQ™ and water to 50 µL. The PCR program was: 5 min, 95 °C; 30 cycles of 1 min, 94 °C, 1 min, 55 °C, 1 min 72 °C, followed by a final extension of 10 min, 72 °C. The PCR products were purified using a combination of ThermoFisher Exonuclease I and Thermo Scientific FastAP Thermosensitive Alkaline Phosphatase, according to the manufacturers’ instructions, and sequenced by SolGent using primers “805R” (5′GACTACCAGGGTATCTAATCC3′) and “1492R”. (5′TACGGYTACCTTGTTACGACTT3′).

### Sequence Analysis

The sequence data were assembled using “DNA Baser” software (version 3) using the ABI sequence format, and the identification of phylogenetic neighbours and calculation of pairwise 16 s rRNA gene sequence similarities was achieved using the EZTaxon-e-server (http://www.ezbiocloud.net/taxonomy) [83]. The resultant sequences were aligned using the CLUSTAL W algorithm from the MEGA 7 software package [84]. Phylogenetic analyses using the maximum-likelihood (ML) algorithm [85] were realised using MEGA 7 software package [84]. The topologies of the resultant tree were evaluated using bootstrap analysis [86] based on 1000 replicates used in conjunction with tree-bisection and reconnection branch swapping and ten additional random sequence replicates for MP and rapid bootstrapping in conjunction with the auto MRE bootstrapping criterion [87]. The tree was rooted using the 16 s rRNA sequence of *Bacillus subtilis* DSM 10(T) ATCC 6051.

## Supplementary Information

Below is the link to the electronic supplementary material.

Fig. S1 Molecular Phylogenetic analysis of all rounded/small S. pombe phenotype inducing strains using the Maximum Likelihood method The evolutionary history was inferred by using the Maximum Likelihood method based on the Tamura-Nei model [88]. The tree with the highest log likelihood (-4089.01) is shown. The percentage of trees in which the associated taxa clustered together is shown next to the branches. Initial tree(s) for the heuristic search were obtained automatically by applying Neighbor-Join and BioNJ algorithms to a matrix of pairwise distances estimated using the Maximum Composite Likelihood (MCL) approach, and then selecting the topology with superior log likelihood value. The tree is drawn to scale, with branch lengths measured in the number of substitutions per site. The analysis involved 143 nucleotide sequences. Evolutionary analyses were conducted using MEGA7 [ 2016].Supplementary file1 (PDF 207 kb)

Fig. S2 Molecular Phylogenetic analysis of candicidin only producing strains by the Maximum Likelihood method The evolutionary history was inferred by using the Maximum Likelihood method based on the Tamura-Nei model [88]. The tree with the highest log likelihood (-3286.00) is shown. The percentage of trees in which the associated taxa clustered together is shown next to the branches. Initial tree(s) for the heuristic search were obtained automatically by applying Neighbor-Join and BioNJ algorithms to a matrix of pairwise distances estimated using the Maximum Composite Likelihood (MCL) approach, and then selecting the topology with superior log likelihood value. The tree is drawn to scale, with branch lengths measured in the number of substitutions per site. The analysis involved 35 nucleotide sequences. All positions containing gaps and missing data were eliminated. Evolutionary analyses were conducted using MEGA7 [2016]. Supplementary file2 (PDF 11 kb)

Fig. S3. Molecular Phylogenetic analysis of S. enissocaesilis related strains identified during the study by Maximum Likelihood method. The evolutionary history was inferred by using the Maximum Likelihood method based on the Tamura-Nei model [88]. The tree with the highest log likelihood (-3070.23) is shown. The percentage of trees in which the associated taxa clustered together is shown next to the branches. Initial tree(s) for the heuristic search were obtained automatically by applying Neighbor-Join and BioNJ algorithms to a matrix of pairwise distances estimated using the Maximum Composite Likelihood (MCL) approach, and then selecting the topology with superior log likelihood value. The tree is drawn to scale, with branch lengths measured in the number of substitutions per site. The analysis involved 18 nucleotide sequences. All positions containing gaps and missing data were eliminated. Evolutionary analyses were conducted using MEGA7 [2016].Supplementary file3 (PDF 60 kb)

Supplementary file4 (DOC 58 kb)

Supplementary file5 (DOCX 13 kb)

Supplementary file6 (DOCX 16 kb)

## Declarations

### Research Involving Human and Animal Participants

This article does not contain any studies with human participants or animals performed by any of the authors.
