# Supplementary figures and images for: Screening and Purification of Natural Products from Actinomycetes that Induce a “Rounded” Morphological Phenotype in Fission Yeast
Source: Nat Prod Bioprospect. 2021 Apr 21;11(4):431–45. doi: 10.1007/s13659-021-00304-1 (PMC8275771; doi:10.1007/s13659-021-00304-1)

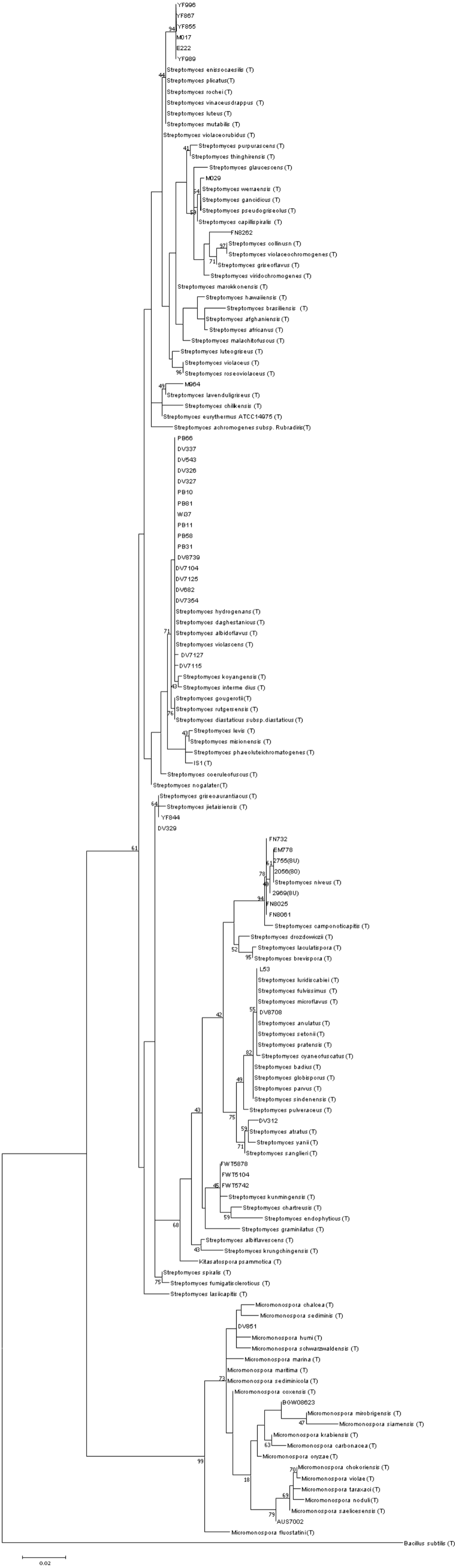

Supplement: Supplementary file 1 — Fig. S1 Molecular Phylogenetic analysis of all rounded/small S. pombe phenotype inducing strains using the Maximum Likelihood method The evolutionary history was inferred by using the Maximum Likelihood method based on the Tamura-Nei model [88]. The tree with the highest log likelihood (-4089.01) is shown. The percentage of trees in which the associated taxa clustered together is shown next to the branches. Initial tree(s) for the heuristic search were obtained automatically by applying Neighbor-Join and BioNJ algorithms to a matrix of pairwise distances estimated using the Maximum Composite Likelihood (MCL) approach, and then selecting the topology with superior log likelihood value. The tree is drawn to scale, with branch lengths measured in the number of substitutions per site. The analysis involved 143 nucleotide sequences. Evolutionary analyses were conducted using MEGA7 [ 2016].Supplementary file1 (PDF 207 kb) [file 13659_2021_304_MOESM1_ESM.pdf]

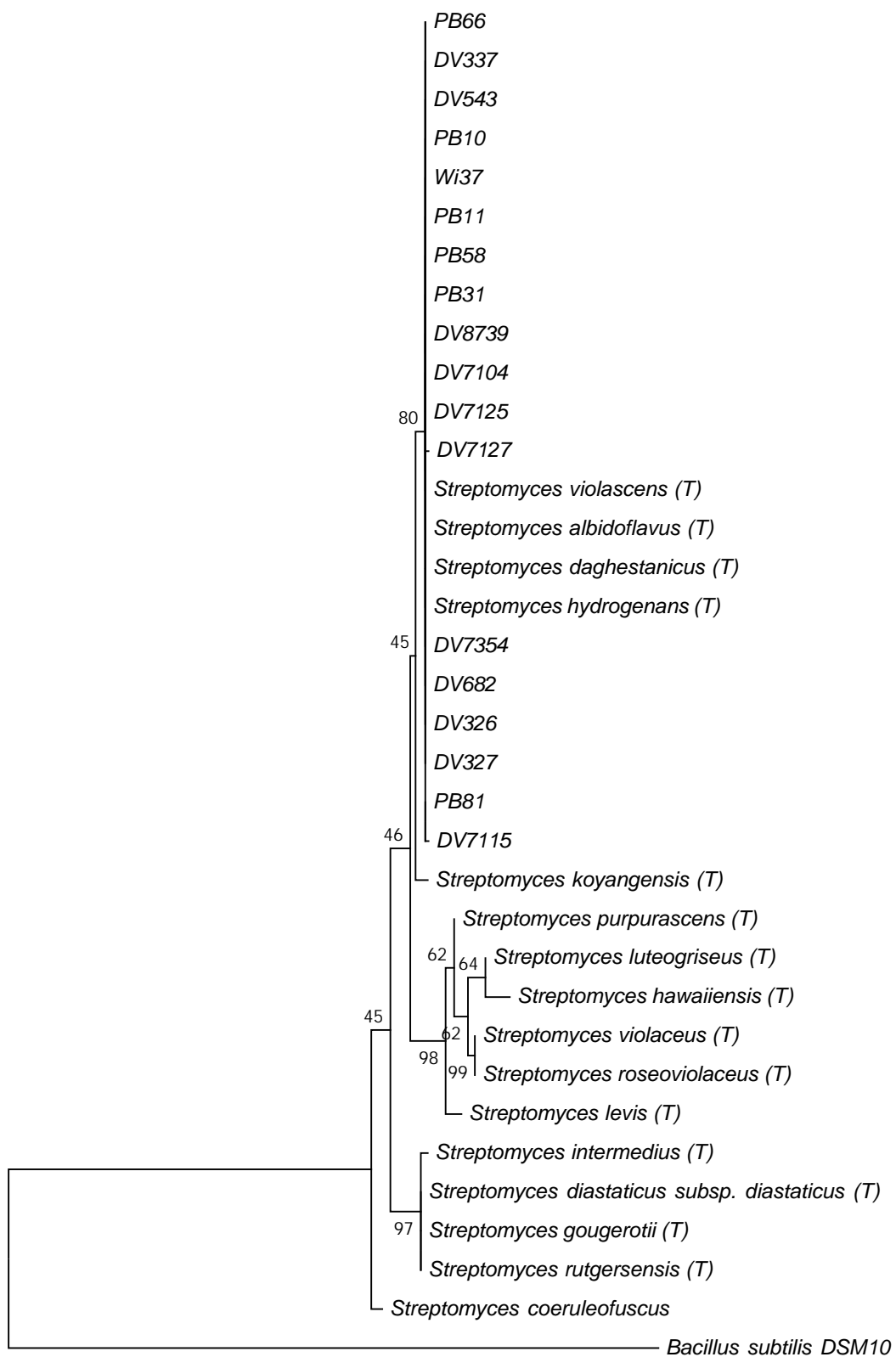

0.020

Supplement: Supplementary file 2 — Fig. S2 Molecular Phylogenetic analysis of candicidin only producing strains by the Maximum Likelihood method The evolutionary history was inferred by using the Maximum Likelihood method based on the Tamura-Nei model [88]. The tree with the highest log likelihood (-3286.00) is shown. The percentage of trees in which the associated taxa clustered together is shown next to the branches. Initial tree(s) for the heuristic search were obtained automatically by applying Neighbor-Join and BioNJ algorithms to a matrix of pairwise distances estimated using the Maximum Composite Likelihood (MCL) approach, and then selecting the topology with superior log likelihood value. The tree is drawn to scale, with branch lengths measured in the number of substitutions per site. The analysis involved 35 nucleotide sequences. All positions containing gaps and missing data were eliminated. Evolutionary analyses were conducted using MEGA7 [2016]. Supplementary file2 (PDF 11 kb) [file 13659_2021_304_MOESM2_ESM.pdf]

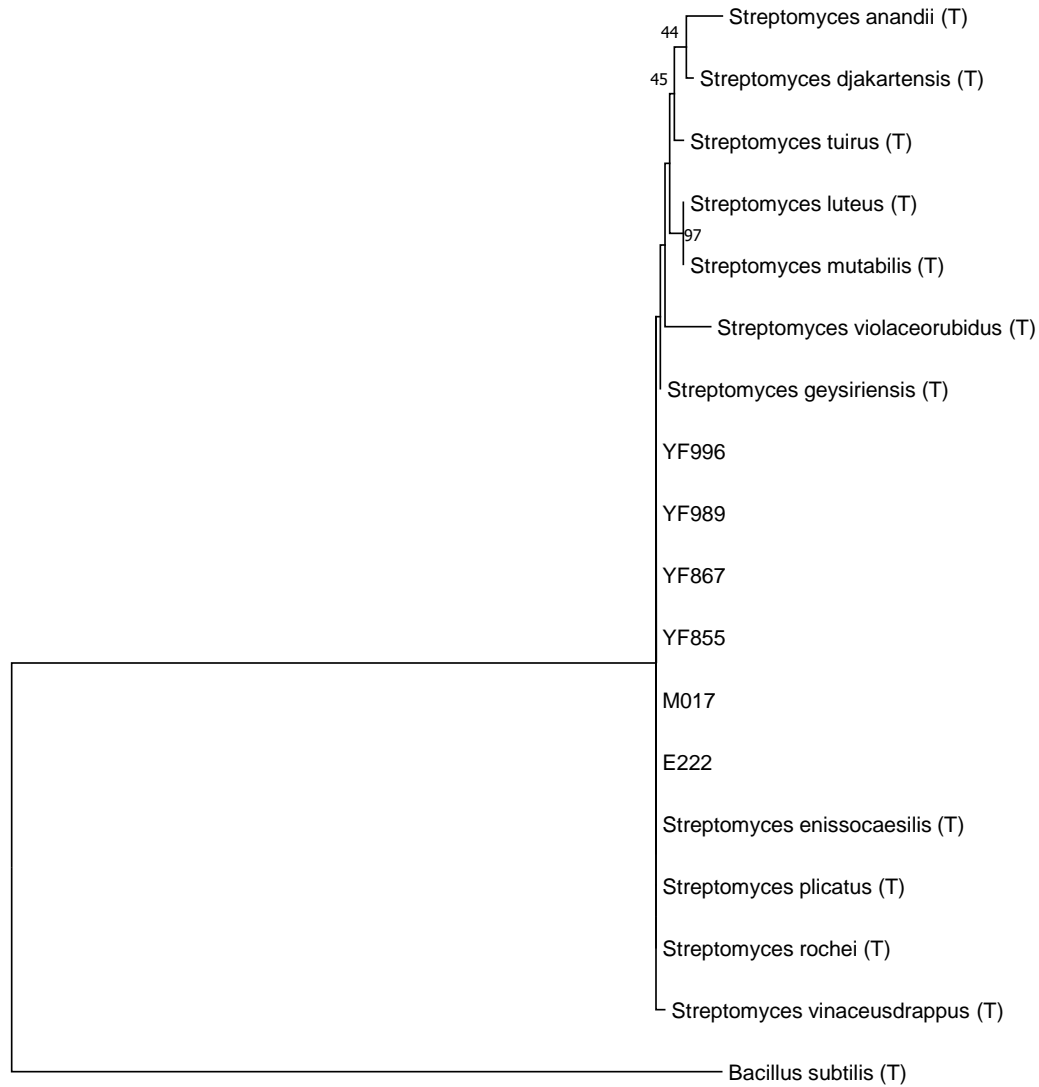

Supplement: Supplementary file 3 — Fig. S3. Molecular Phylogenetic analysis of S. enissocaesilis related strains identified during the study by Maximum Likelihood method. The evolutionary history was inferred by using the Maximum Likelihood method based on the Tamura-Nei model [88]. The tree with the highest log likelihood (-3070.23) is shown. The percentage of trees in which the associated taxa clustered together is shown next to the branches. Initial tree(s) for the heuristic search were obtained automatically by applying Neighbor-Join and BioNJ algorithms to a matrix of pairwise distances estimated using the Maximum Composite Likelihood (MCL) approach, and then selecting the topology with superior log likelihood value. The tree is drawn to scale, with branch lengths measured in the number of substitutions per site. The analysis involved 18 nucleotide sequences. All positions containing gaps and missing data were eliminated. Evolutionary analyses were conducted using MEGA7 [2016].Supplementary file3 (PDF 60 kb) [file 13659_2021_304_MOESM3_ESM.pdf]
