# Supplementary material for: Screening and Purification of Natural Products from Actinomycetes that Induce a “Rounded” Morphological Phenotype in Fission Yeast
Source: Nat Prod Bioprospect. 2021 Apr 21;11(4):431–45. doi: 10.1007/s13659-021-00304-1 (PMC8275771; doi:10.1007/s13659-021-00304-1)
Supplement: Supplementary file 4 — Supplementary file4 (DOC 58 kb) [file 13659_2021_304_MOESM4_ESM.doc]

| **Strain name** | **No of nucleotides different from closest species match** | **Closest 16srRNA species match** | **Natural products produced** |
| --- | --- | --- | --- |
| DV682 | 0/1402 | *Streptomyces hydrogenans* | Candicidin |
| DV7354 | 0/1408 | *Streptomyces hydrogenans* | Candicidin |
| DV7115 | 1/1421 | *Streptomyces hydrogenans* | Candicidin |
| DV7125 | 0/1424 | *Streptomyces hydrogenans* | Candicidin |
| DV8739 | 0/1419 | *Streptomyces hydrogenans* | Candicidin |
| PB31 | 0/1423 | *Streptomyces hydrogenans* | Candicidin |
| PB66 | 0/1420 | *Streptomyces hydrogenans* | Candicidin |
| PB58 | 0/1423 | *Streptomyces hydrogenans* | Candicidin |
| PB11 | 0/1432 | *Streptomyces hydrogenans* | Candicidin |
| PB81 | 0/1420 | *Streptomyces hydrogenans* | Candicidin |
| PB10 | 0/1409 | *Streptomyces hydrogenans* | Candicidin |
| DV327 | 0/1420 | *Streptomyces hydrogenans* | Candicidin |
| DV326 | 0/1425 | *Streptomyces hydrogenans* | Candicidin |
| DV337 | 1/1420 | *Streptomyces hydrogenans* | Candicidin |
| Wi37 | 0/1409 | *Streptomyces albidoflavus* | Candicidin |
| DV7104 | 2/1415 | *Streptomyces albidoflavus* | Candicidin |
| DV543 | 2/1416 | *Streptomyces albidoflavus* | Candicidin |
| DV7127 | 3/1424 | *Streptomyces albidoflavus* | Candicidin |
| M017 | 0/1420 | *Streptomyces enissocaesilis* | Candicidin |
| E222 | 0/1423 | *Streptomyces enissocaesilis* | Candicidin  Streptothricin |
| YF867 | 0/1418 | *Streptomyces enissocaesilis* | Candicidin  Streptothricin |
| YF989 | 0/1424 | *Streptomyces enissocaesilis* | Candicidin  Streptothricin |
| YF996 | 0/1419 | *Streptomyces enissocaesilis* | Candicidin  Streptothricin |
| YF855 | 0/1418 | *Streptomyces enissocaesilis* | Candicidin  Streptothricin |
| IS1 | 3/1414 | *Streptomyces misionensis* | Fungichromin |
| DV329 | 6/1402 | *Streptomyces spiralis* | Filipin |
| 2969(8U) | 0/1425 | *Streptomyces niveus* |  |
| 2755(8U) | 0/1221 | *Streptomyces niveus* |  |
| EM778 | 2/1427 | *Streptomyces niveus* |  |
| 2056(8U) | 3/1407 | *Streptomyces niveus* |  |
| FN8061 | 5/721 | *Streptomyces niveus* |  |
| FWT5742 | 7/1407 | *Streptomyces kunmingensis* |  |
| FWT5878 | 7/1399 | *Streptomyces kunmingensis* |  |
| FWT5104 | 7/1409 | *Streptomyces kunmingensis* |  |
| FN8025 | 0/1419 | *Streptomyces camponoticapitis* |  |
| FN732 | 7/1417 | *Streptomyces camponoticapitis* |  |
| FN8262 | 9/1412 | *Streptomyces collinus* |  |
| L53 | 0/1339 | *Streptomyces cyaneofuscatus* |  |
| DV8708 | 0/1425 | *Streptomyces anulatus* |  |
| M029 | 0/1420 | *Streptomyces werranesis* |  |
| M964 | 2/1352 | *Streptomyces lavenuligriseus* |  |
| DV312 | 3/1424 | *Streptomyces atratus* |  |
| YF844 | 0/1408 | *Streptomyces griseoauranticus* |  |
| BGW08623 | 10/1406 | *Micromonospora krabiensis* |  |
| DV851 | 4/1344 | *Micromonospora humi* |  |
| AUS7002 | 7/1436 | *Micromonospora chokoriensis* |  |
