# Supplementary material for: Screening and Purification of Natural Products from Actinomycetes that Induce a “Rounded” Morphological Phenotype in Fission Yeast
Source: Nat Prod Bioprospect. 2021 Apr 21;11(4):431–45. doi: 10.1007/s13659-021-00304-1 (PMC8275771; doi:10.1007/s13659-021-00304-1)
Supplement: Supplementary file 5 — Supplementary file5 (DOCX 13 kb) [file 13659_2021_304_MOESM5_ESM.docx]

| **Strain name** | **No of nucleotides different from closest species match** | **Closest 16srRNA species match** | **Natural products produced** |
| --- | --- | --- | --- |
| A62 | 0/1428 | *Streptomyces hydrogenans* | Candicidin |
| MR620 | 0/1405 | *Streptomyces hydrogenans* | Candicidin |
| MDA8-444 | 0/1427 | *Streptomyces hydrogenans* | Candicidin |
| WAB680 | 2/1405 | *Streptomyces hydrogenans* | Candicidin |
| S34 | 4/1428 | *Streptomyces hydrogenans* | Candicidin |
| MU147-B | 8/1436 | *Streptomyces hydrogenans* | Candicidin |
| MU1858 | 9/1434 | *Streptomyces albidoflavus* | Candicidin |
| MU1476 | 9/1434 | *Streptomyces albidoflavus* | Candicidin |
| E223 | 0/1405 | *Streptomyces plicatus* | Candicidin, Streptothricin |
| B12 | 0/1413 | *Streptomyces mutabilis* | Candicidin |
| 5231 T (12) | 0/1448 | *Streptomyces rochei* | Candicidin |
