# Supplementary material for: Screening and Purification of Natural Products from Actinomycetes that Induce a “Rounded” Morphological Phenotype in Fission Yeast
Source: Nat Prod Bioprospect. 2021 Apr 21;11(4):431–45. doi: 10.1007/s13659-021-00304-1 (PMC8275771; doi:10.1007/s13659-021-00304-1)
Supplement: Supplementary file 6 — Supplementary file6 (DOCX 16 kb) [file 13659_2021_304_MOESM6_ESM.docx]

**Supplementary Figure captions**

**Fig. S1**

**Molecular Phylogenetic analysis of all rounded/small *S. pombe* phenotype inducing strains using the Maximum Likelihood method**

The evolutionary history was inferred by using the Maximum Likelihood method based on the Tamura-Nei model [88]. The tree with the highest log likelihood (-4089.01) is shown. The percentage of trees in which the associated taxa clustered together is shown next to the branches. Initial tree(s) for the heuristic search were obtained automatically by applying Neighbor-Join and BioNJ algorithms to a matrix of pairwise distances estimated using the Maximum Composite Likelihood (MCL) approach, and then selecting the topology with superior log likelihood value. The tree is drawn to scale, with branch lengths measured in the number of substitutions per site. The analysis involved 143 nucleotide sequences. Evolutionary analyses were conducted using MEGA7 [ 2016].

**Fig. S2**

**Molecular Phylogenetic analysis of candicidin only producing strains by the Maximum Likelihood method**

The evolutionary history was inferred by using the Maximum Likelihood method based on the Tamura-Nei model [88]. The tree with the highest log likelihood (-3286.00) is shown. The percentage of trees in which the associated taxa clustered together is shown next to the branches. Initial tree(s) for the heuristic search were obtained automatically by applying Neighbor-Join and BioNJ algorithms to a matrix of pairwise distances estimated using the Maximum Composite Likelihood (MCL) approach, and then selecting the topology with superior log likelihood value. The tree is drawn to scale, with branch lengths measured in the number of substitutions per site. The analysis involved 35 nucleotide sequences. All positions containing gaps and missing data were eliminated. Evolutionary analyses were conducted using MEGA7 [2016].

**Fig. S3.**

**Molecular Phylogenetic analysis of *S. enissocaesilis* related strains identified during the study by Maximum Likelihood method.**

The evolutionary history was inferred by using the Maximum Likelihood method based on the Tamura-Nei model [88]. The tree with the highest log likelihood (-3070.23) is shown. The percentage of trees in which the associated taxa clustered together is shown next to the branches. Initial tree(s) for the heuristic search were obtained automatically by applying Neighbor-Join and BioNJ algorithms to a matrix of pairwise distances estimated using the Maximum Composite Likelihood (MCL) approach, and then selecting the topology with superior log likelihood value. The tree is drawn to scale, with branch lengths measured in the number of substitutions per site. The analysis involved 18 nucleotide sequences. All positions containing gaps and missing data were eliminated. Evolutionary analyses were conducted using MEGA7 [2016].

**Table S1**

Table providing a list of strains investigated in the present study which induce a small/rounded *S. pombe* phenotype and the corresponding 16s rRNA sequencing data, the most closely related described named species and natural products which they synthesize, as determined during the course of the study.

**Table S2**

Table providing a list of additional strains investigated in the present study (which were not previously known to induce a small/rounded *S. pombe* phenotype) and the corresponding 16s rRNA sequencing data, the most closely related described named species and natural products which they synthesize, as determined during the course of the study.
